# Multifaceted Factors Causing Conflicting Outcomes in Herb-Drug Interactions

**DOI:** 10.3390/pharmaceutics13010043

**Published:** 2020-12-30

**Authors:** Young Hee Choi, Young-Won Chin

**Affiliations:** 1 College of Pharmacy and Integrated Research Institute for Drug Development, Dongguk University_Seoul, 32 Dongguk-lo, Ilsandong-gu, Goyang-si, Gyeonggi-do 10326, Korea; 2 College of Pharmacy and Research Institute of Pharmaceutical Sciences, Seoul National University, Seoul 08826, Korea; ywchin@snu.ac.kr

**Keywords:** herb-drug interaction, pharmacokinetics, dose, treatment period

## Abstract

Metabolic enzyme and/or transporter-mediated pharmacokinetic (PK) changes in a drug caused by concomitant herbal products have been a primary issue of herb and drug interactions (HDIs), because PK changes of a drug may result in the alternation of efficacy and toxicity. Studies on HDIs have been carried out by predictive in vitro and in vivo preclinical studies, and clinical trials. Nevertheless, the discrepancies between predictive data and the clinical significance on HDIs still exist, and different reports of HDIs add to rather than clarify the confusion regarding the use of herbal products and drug combinations. Here, we briefly review the underlying mechanisms causing PK-based HDIs, and more importantly summarize challenging issues, such as dose and treatment period effects, to be considered in study designs and interpretations of HDI evaluations.

## 1. Introduction

The use of herbal products, including herbal medicines and dietary supplements, is increasing, and the concurrent use of herbal products with drugs is also accelerating [1]. Herb-drug (HD) combinations can result in unexpected effects (i.e., efficacy loss or increased toxicity) due to the interference of pharmacological effect(s) between herbal products and drugs, which are defined as herb-drug interactions (HDIs) [2,3]. Given the many HDI cases that have already been reported (e.g., those featuring St. John’s Wort, ginkgo, or kava) [4,5,6], it has become evident that HDIs are strongly associated with changes of the pharmacokinetics (PK) of a drug, which are caused by the co-administration of a herbal product [2,3,4]. In other words, changes to the PK of a drug (e.g., warfarin, insulin, aspirin, digoxin, or cyclosporine) by herbal products are mediated mainly by the inhibition or induction of metabolic enzymes and/or transporters, and this may also consequently cause changes in the pharmacological action [4,7,8]. When a herbal product as a perpetrator modulates metabolic enzymes and/or transporters impacting the PK of a drug, plasma and/or tissue concentrations of the drug are altered, thereby leading to unexpected changes in the pharmacological or toxicological effects [4,9].

To evaluate PK-based HDIs in HD combinations, various assay systems (e.g., in vitro, or in vivo preclinical and clinical studies) have been developed [3,10,11,12,13]. Regulatory agencies such as the U.S. Food and Drug Administration (FDA) and the European Medicines Agency (EMA) have suggested guidelines for HDI studies, and recommend the evaluation of PK-based HDIs under development and on the market [3,4]. Moreover, detailed information on the potential effects of HDIs as mediated by metabolic enzymes and transporters is required, and currently some knowledge of HDIs is available at the time of market approval [2,5,14]. Despite the accumulation of scientific knowledge which has contributed to the understanding of mechanisms behind HDIs, inconsistent predictions and/or results from HDIs still exist due to the complex nature of herbal products (e.g., multiple and complex chemicals in herbal products), assay systems (e.g., in vitro, or in vivo preclinical studies or clinical cases) and/or diverse factors considered in study designs (e.g., dose and treatment periods) [3,10,15,16]. Moreover, imprudent interpretations or judgments for PK-based HDIs without a full understanding of study designs make it difficult to determine the proper use of HD combination. 

Here, we present a brief overview of metabolism- and transporter-mediated pathways relevant to HDIs and further discuss the causes of conflicting predictions and results of HDIs. Furthermore, we discuss challenging issues and viewpoints when designing evaluation systems and interpreting outcomes of HDIs.

## 2. Main Pathways Causing HDIs: Metabolic Enzyme- and Transporter-Mediated HDIs

**Figure 1:**
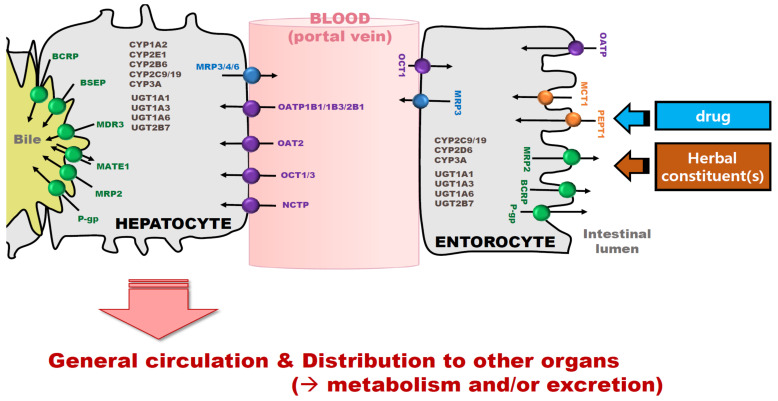
Transporter- and metabolic enzyme-mediated disposition patterns of drug and herbal products.

Herbal products utilize the same metabolic pathways and transporter systems as drugs during the absorption, distribution, metabolism and/or excretion (ADME) processes in the body (Figure 1). Tsai et al. [4] reported that approximately 43% of HDI cases were related to PK-based interactions, and even contraindication cases resulting from HD combinations occurred. In the evaluation of PK-based drug-drug interactions (DDIs), a drug of interest has served as either a substrate (a victim drug) or an inhibitor or inducer (a perpetrator drug) for metabolic enzymes and/or transporters [9,17]. Co-administered drugs in DDIs can alter the PK of a victim drug, and evaluating DDI between a victim drug and a perpetrator drug is recommended [9,18,19]. However, herbal products of interest are generally assumed to be perpetrators (i.e., an inhibitor or inducer), whereas a drug is often considered to be a substrate (a victim drug) for metabolic enzymes or transporters when evaluating HDIs. This comes from the fact that most herbal products contain a mixture of numerous chemical constituents and sometimes even unknown compounds [20,21,22]. It is not easy to analyze the concentration changes of all chemicals in herbal products representing their PK properties, therefore herbal products are primarily considered to be perpetrators in many PK-based HDI evaluations [1,15,21,22,23]. In addition, most pharmacological effects of a drug as a single treatment are well known, and the changes to the drug by co-administered herbal product can definitely be clarified. Hence, HDI evaluations usually deal with the PK and pharmacodynamic (PD) changes of a drug used in an HD combination [3].

Drugs or chemicals in herbal products entering into the tissues across a membrane (e.g., apical membrane of enterocytes, sinusoidal membrane in hepatocytes, or basolateral membrane of renal proximal epithelial cells) are regulated by passive diffusion and/or transporters. Then, drugs and chemicals from herbal products are cleared by metabolism through phase I and II metabolic enzymes and/or transporter-mediated excretion (e.g., renal or biliary excretion) [24,25,26]. Specifically, bioavailability, an important PK parameter, is variable as a result of the first pass effect including intestinal uptake and efflux transporters, intestinal metabolizing enzymes, and metabolism from hepatic uptake. In comparison, the changes in distribution, metabolism, and excretion mainly occur through modulation of uptake and efflux transporters and/or inhibition/induction of metabolizing enzymes in the respective tissues. These ADME pathways determine the extent of plasma or tissue exposure to herbal products and/or drugs, thereby accounting for the efficacy and toxicity of HD combinations [11,17,27,28].

As underlying mechanisms causing PK-based HDIs [4,9,29,30], first, herbal products affect transporter or metabolic enzyme, respectively. Inhibition of transporters by herbal products in the apical membrane of enterocytes leads to decreased transporter-mediated efflux of a drug and resultantly increases the absorption of a drug (e.g., ginkgo and milk thistle) [3,31]. Milk thistle is a herbal product that acts as a P-gp inhibitor (P-gp) in the liver and kidney, and is able to alter the biliary excretion and renal excretion of a drug [3,31,32] and consequently increase systemic exposure to a drug by increasing plasma concentration [3]. Additionally, herbal products inhibit or induce metabolic enzymes that influence the elimination of a co-administered drug along with systemic exposure change. The controversial effect (i.e., inhibitory and inductive effects) of herbal products on specific transporter or metabolic enzyme have been reported [33]. Chronic co-administration of St. John’s Wort extract reduced the plasma concentration of midazolam via the induction of CYP 3A by St. John’s Wort, but a single administration of St. John’s Wort extract inhibited CYP 3A4 [33].

Secondly, herbal products simultaneously affect transporter and metabolic enzymes; transporters and metabolic enzymes altered by herbal products sometimes show synergistic potential [34]. Some herbal products (e.g., garlic, ginkgo, ginseng, and grape juice) [3,4,31,35] function as both P-gp inhibitors and CYP3A inhibitors. It was reported that both P-gp and CYP3A inhibition by herbal product in enterocytes enhanced systemic exposure to orally administered drug [34]. Milk thistle inhibits P-gp mediated efflux and CYP3A-mediated metabolism of an orally administered cyclosporin A in the intestines, thereby increasing the oral bioavailability of cyclosporine A [9,34]. As another example, herbs can inhibit P-gp in the liver and reduce drug efflux from the liver into the bile, while reducing CYP3A-mediated metabolism of a drug as a CYP3A inhibitor, resulting in increased drug concentrations in the liver and plasma [35]. Co-administered phytochemicals such as piperine or capsaicin with doxorubicin inhibit the biliary excretion and hepatic metabolism of doxorubicin, and consequently, doxorubicin concentrations in the liver and plasma were increased [36].

More interestingly, herbal products can affect transporter and metabolic enzyme-mediated PK properties of parent drug and metabolites. When *Rhodiola rosea* was co-administered with losartan, *R. rosea* increased plasma concentrations of losartan due to CYP2C9 and P-gp inhibition leading to increased bioavailability of losartan. Interestingly, the plasma concentration of EXP-3174, an active metabolite of losartan, was increased despite the lessened formation of EXP-3174 from losartan due to the CYP2C9 inhibition by *R. rosea*. This could be due to that the inhibitory effect of *R. rosea* on CYP2C9-mediated metabolism was stronger on EXP-3174 than losartan [37,38].

Considering that the intestine, liver and kidneys are known as the main organs expressing transporters and metabolic enzymes [17,18], transporter and metabolic enzyme-mediated PK changes in a drug caused by herbal products in the intestine, liver and kidneys have been the primary focus in the evaluations of HDIs.

## 3. Challenging Issues in the Evaluation and Interpretation of PK-Based HDIs

The assessment of HDIs comprises combinations of in vitro and in vivo studies that aim to identify PK interactions. In these studies, in vitro screens are generally followed by dedicated in vivo preclinical and clinical studies [15,20]. The methodologies used to evaluate PK-based HDIs have been upgraded, and the large numbers of reports have verified HDIs; however, the outcomes of these HDI studies may not cover every single permutation of HD combination. For some herbal materials, the incidences of HDIs still happen in clinical cases. Moreover, the conflicting HDI outcomes in the literature makes it confusing and difficult to predict the extent or clinical significance of HDIs [15]. Thus, we highlight the challenges when evaluating and interpreting PK-based HDIs and propose viewpoints when designing studies to analyze PK-based HDIs and interpreting inconsistent HDI evaluation outcomes. Three reasons are suggested to be causes of diverse outcomes in HDI evaluations and inappropriate interpretation of HDI outcomes. These reasons are: (1) the complex nature of herbal products; (2) responses of drugs’ and/or herbs’ exposure to different assay systems (e.g., in vitro and in vivo); and (3) diverse factors in study designs (e.g., dose, treatment period, administration route, etc.) [15].

### 3.1. The Complex Nature of Herbal Products

Herbal products are used as a single extract or complex extracts containing multiple components [39,40]. The chemical constituents in a herb or herbal extract made from a plant with the same binomial name can vary with the cultivation area, harvest time, storage condition, and extraction methods [41,42,43,44]. For these reasons, it is not easy to prepare herbal extracts or herbal preparations with similar or identical chemical compositions. In other words, it is very possible that differences in the quality of herbal extracts used by different research groups in HDI evaluations may be different; therefore, HDI evaluations using herbal preparations made from plant materials with the same binomial name may end with conflicting results. Other changes, including adulteration, misidentification, contamination, or substitution, to the properties of herbal preparations may also occur [40,45]. Moreover, given that herbal products contain various bioactive compounds, the results of HDI studies based on pure active constituents can be inconsistent with the HDI results acquired by exploring the herbal product itself (i.e., a herbal extract). For example, unknown constituents in a herbal product may modulate cytochrome P450s (CYPs), but their amount and inhibition/induction potency against CYPs cannot be predicted. Only the overall effect of a herbal product on the modulation of CYPs has been explored [46]. All these factors can contribute to the conflicting overall HDI observations especially in herbal extracts used in HD combinations [40,42,47]. Therefore, when reporting HDI outcomes, binomial names and parts of the plant used in the herbal preparations, extraction methods and chemical composition of the herbal preparations (e.g., chemical and bio-response fingerprints among differently manufactured batches) should be provided [43,44].

### 3.2. Responses of a Drug’s or Herbal Product’s Exposure to Different Assay Systems 

Inconsistent responses from a drug’s and/or herbal product’s exposure to various assay systems (e.g., in vitro or in vivo preclinical and clinical studies) can occur. The potential of drug interactions (e.g., DDI and HDI) is primarily evaluated through inhibition/ induction abilities of metabolism in in vitro assay systems (e.g., recombinant CYPs/uridine 5′-diphospho-glucuronosyltransferase-glucuronosyltransferase (UGTs), microsomes, cytosol, S9 fraction, cell lines, transgenic cell lines, primary or cryopreserved hepatocytes) [13,48]. Data from in vitro assay systems provide potential mechanisms that can cause PK-based drug interactions, which are based on widespread in vivo preclinical studies, clinical PK, and clinical reports [12,13,15,49]. These assay systems have been applied to most drug interaction studies such as DDI and HDI. Especially, when herbal product only influences the metabolic pathway and this metabolic pathway is primary property in the disposition of co-administered drug, in vitro metabolism studies are important for predicting clinical HDIs through extrapolating in vitro data to humans [46].

Although many in vitro systems mentioned above have been effectively used to predict HDIs, in vitro assay systems sometimes show intrinsic drawbacks explaining the underlying mechanisms causing PK-based HDIs [13,48], and challenging issues in HDI evaluation that use various in vitro assay systems are of concern. For example, liver microsomes, which are widely used in in vitro assay system to evaluate metabolic interactions, do not represent the true in vivo situation because only the endoplasmic reticulum-localized enzymes are contained in liver microsomes, and metabolic interactions via other enzymes cannot be detected [48,50]. The concurrent interaction via transporters and metabolic enzymes are involved in in vivo ADME process, therefore one targeted metabolic enzyme or transporter in in vitro screening is not enough nor even related to the in vivo phenomena [9,31,51]. Furthermore, orally administered ginsenosides affect hepatic CYPs, and their metabolites also alter intestinal P-gp in vivo [52,53]. However, the effects of ginsenosides and their metabolites on metabolic enzymes or transporter-mediated interactions cannot be simultaneously investigated in an in vitro assay system. When the effects of ginsenosides on CYPs in in vitro human microsome or recombinant CYPs and P-gp in in vitro Caco-2 cells, respectively, have been evaluated [53], their quantitative contributions to in vivo HDI outcomes of individual ginsenosides cannot be calculated, implying it is impractical to predict HDIs based only on in vitro studies. Thus, in vivo preclinical systems are necessary to assess HDI outcomes by considering the dispositions of herbal products and drugs (i.e., simultaneous modulation of metabolizing enzymes and transporters by herbal products influencing drug disposition). Although in vivo preclinical studies are important and required to avoid the occurrence of serious adverse reactions of HD combination at clinical levels [46], conflicting outcomes between in vivo preclinical and clinical results still exist [15], due to interspecies variability especially between primates and rodents [51,54]. For example, interspecies variability in metabolizing enzymes [55] and transporters, such as P-gp [55,56] renal organic anion transporters (OATs) and organic cation transporters (OCTs) [57], may contribute to contradictory HDI results [15]. In addition, the dose and/or treatment period of in vivo preclinical studies sometimes do not reflect clinically recommended dosage regimens, and the extrapolation of dosage regimens between in vivo preclinical and clinical data should be considered.

In addition, the outcomes of HDIs among different assay systems can fluctuate as a result of the multiple components in herbal extracts [58]. Multiple constituents in herbal extracts can interact or not interact individually with specific metabolic enzymes and transporters, which can also affect the PK changes to a co-administered drug. The unabsorbed constituents in orally administered herbal extracts are not involved in in vivo interactions in the hepatic metabolism of a co-administered drug [15,59]. However, all constituents in an herbal extract, even some constituents that do not reach the liver due to the lack of absorption in in vivo systems, are treated with a drug together in in vitro systems using hepatocytes and liver microsomes. Hence, inconsistent HDI data between in vitro and in vivo systems can occur. 

### 3.3. Multifaceted Factors in Study Designs (E.g., Administration Route, Dose, Treatment Period, Etc.)

**Figure 2:**
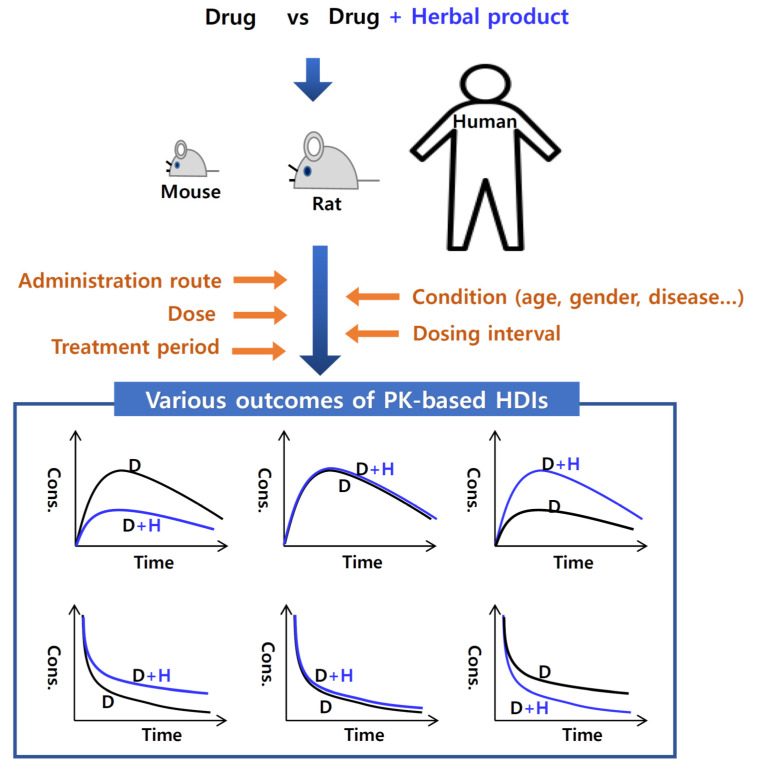
Schematic illustration of the factors that influence herb–drug interactions (HDIs) and the potential outcomes of pharmacokinetic (PK) interactions. The upper three graphs represent plasma concentration-time curve of a drug after oral administration of a drug with and without herbal product: left, co-administered herbal product decreases the plasma concentration of a drug; middle; co-administered herbal product does not affect the plasma concentration of a drug; right; co-administered herbal product increases the plasma concentration of a drug. Also the lower three graphs represent plasma concentration-time curve of a drug after intravenous administration of a drug with and without herbal product: left, co-administered herbal product increases the plasma concentration of a drug; middle; co-administered herbal product does not affect the plasma concentration of a drug; right; co-administered herbal product decreases the plasma concentration of a drug. In all cases, a herbal product is orally administered. D and H indicates a drug and a herbal product, respectively.

The administration route, dose, treatment period, and probe chemicals (i.e., substrates, inhibitors, and inducers) are critical factors that determine whether HDIs occur or not, which can cause inconsistent HDI examination results [15]. Therefore, a diverse number of factors should be elaborately estimated and considered when explaining the outcomes of HDI studies (Figure 2 and Table 1).

Firstly, administration routes of both herbal products and drug should be considered. Considering that most herbal products are orally administered in clinical cases, intestinal metabolism-mediated interactions are important, especially when a drug is also orally administered [60]. In particular, HDIs in the intestinal sites would also be clinically relevant and should be examined carefully, because both herbal products and drugs are orally administered in most cases, and an abundant number of metabolizing enzymes and transporters are expressed in the intestine [15]. For example, orally administered *Ginkgo biloba* leaf extracts only alter PK when nifedipine is administered orally, but not intravenously, in rats [61]. Orally administered *Zingiber officinale* root juice decreases the oral bioavailability of cyclosporine, but the PK of intravenous cyclosporine is not altered [62]. This phenomenon may be due to the different modulatory effects of the herb on metabolic enzymes in liver and intestine [60,63,64,65]. Additionally, *Schisandra chinensis* fruit extract possesses more intensively modulatory effects on intestinal CYP3A than on hepatic CYP3A [64].

Secondly, depending on the administration doses of herbal products and drugs, HDI outcomes are differently produced [15]. Herbal products usually exhibit dose-dependent inductive [66,67,68] or inhibitory [69,70] effects on CYPs, and interestingly they can sometimes have biphasic effects on CYPs. For example, induction occurs at low dosages and inhibition occurs at higher dosages of herbal products [71]. Pre-treatment with a low dose of *Andrographis paniculata* extract increases theophylline elimination due to the induction of CYP1A2, whereas a high dose of *A. paniculata* decreases theophylline elimination due to the inhibition of CYP1A2, respectively [72]. In the case of co-administration of *Tinospora cordifolia* aqueous-alcoholic extract and glibenclamide, only high doses (400 mg/kg) *T. cordifolia* aqueous alcoholic extract, not low doses (100 mg/kg), increased the oral bioavailability of glibenclamide due to the reduced clearance via CYP 2C9, 2D6 and 3A4 in rats [73].

Thirdly, treatment periods may affect the occurrence of HDIs. Long-term treatments with herbal products are common, therefore the effects of herbal products on metabolic enzymes and/or transporters usually appear differently based on the treatment period. For example, short-term treatments with *S. chinensis* fruit extract exerts inhibitory effects, but long-term treatment shows inductive effects on both hepatic and intestinal CYP3A [64]. A single dose of *Glycyrrhiza glabra* root extract does not affect CYPs, although repeated treatments induce hepatic CYP3A, and to a lesser extent, 2B1 and 1A2 in mice [74]. *G. biloba* leaf extract usually shows inhibitory effects on the metabolism of most co-administered drugs [61,75], but reversely its long-term pre-treatment induces hepatic metabolizing activity [68,76]. In addition, Han et al [29] reported that metformin concentrations in the liver were increased as a result of the reduction in mate1-mediated biliary excretion of metformin in rats simultaneously treated with metformin and *Lonicera japonica* extract for a 28-day treatment, not a single or 7-day treatment. They also observed an enhancement of metformin’s glucose tolerance activity only with 28-day treatment period. As another example, You et al. [30] reported that the area under the plasma concentration–time curve (AUC) of metformin was increased due to the decrease in renal oct2-mediated renal excretion of metformin, and metformin concentration in the kidneys was increased as a result of the increase in oct1-mediated renal uptake of metformin along with the enhancement of the glucose-lowering effect in rats that had undergone a 28-day co-treatment of metformin and *Houttuynia cordata* extract. These interactions did not occur in rats that had undergone a single and 7-day co-treatment of metformin and *H. cordata* extract. Thus, choosing the appropriate treatment period is important for HDI evaluations [15].

## 4. Future Perspectives and Conclusion

Substantial progress has been made in the methods used to clinically assess PK-based HDIs, but there are still demands for well-designed clinical trials that will improve our understanding of the underlying mechanisms of HDIs. Additionally, it is important to appropriately communicate the clinical relevance and implications of respective findings, thereby ultimately enabling the continuous improvement of informed clinical decisionmakers when it comes to making HD combinations. Furthermore, assessments of HDI potential and clinically relevant research have been continuously developed and conducted to reduce risks and avoid undesired consequences in HD combinations. In terms of improving the evaluation methodologies and interpretations of HDIs, it is helpful to understand the complicated nature of herbal products, different intrinsic characteristics existing in respective assay systems (i.e., in vitro or in vivo preclinical and clinical studies) and diverse factors considered in study designs. 

In addition, it is necessary to consider the physiological and pathological condition (e.g., intestinal bacteria, underlying diseases, and genetic factors) of patients for the appropriate use of HD combinations [78,79]. Nevertheless, it is challenging to incorporate these various factors into the evaluation of HDIs at the clinical levels. Thus, to extrapolate HDI studies from in vivo preclinical data to humans, several efforts have been attempted: (1) genetically modified animals that have been transfected with human genes to express exactly the same enzymes as humans (e.g., humanized mice) are now available, and these animals closely represent human conditions; and (2) simulation of clinical HDIs are now performed using in vitro and in vivo preclinical data. Given the simulations of HDIs from preclinical data to human cases, PK–PD modeling systems have emerged as useful tools to predict HDI outcomes [80,81]. In addition, optimized dosage regimens of HD combinations that account for the concepts of synergism or antagonism in HD combinations such as drug-drug combinations are required [81,82]. The simulation of HDI outcomes with various dosage regimens using PK–PD modeling may provide a rationale behind choosing the proper HD combinations. In future, computer-assisted or artificial intelligence-guided predictions of extensive HDI outcomes focusing on not only the occurrence of toxicity but also efficacy changes (e.g., synergism and antagonism) caused by diverse factors may be helpful facilitating the evaluation and interpretation of HDI cases [14,83,84,85].

## Figures and Tables

**Table 1:** Factors causing conflicting HDI outcomes.

Factors	Herbal Products	HDI Results	PK-Based HDI Mechanism	Ref
**Administration route**	*Ginkgo biloba* leaf extract (oral, p.o.)	*G. biloba* leaf extract only alters the PK of orally, but not intravenously, administered nifedipine in rats	Due to inhibition of CYP3A in intestine, not in liver	[61]
	*Zingiber officinale* root juice (p.o.)	*Z. officinale* juice decreases the oral bioavailability of cyclosporine, but the PK property of intravenous cyclosporine is not altered in rats	Due to inhibition of CYP3A and P-gp in intestine, not in liver	[62]
	*Echinacea purpurea* root (p.o.)	*E. purpurea* root extract reduced systemic clearance of midazolam following intravenous administration, but oral clearance of midazolam was not altered in rats	Due to inhibition of CYP3A in liver, not in intestine	[77]
	Ginseng berry extract (p.o.)	Ginseng berry extract did not affect the PK properties of intravenous administered nifedipine or cyclosporin, but markedly increased the absolute bioavailability of both drugs after oral administration in rats	Due to inhibition of CYP3A in intestine, not in liver	[77]
	*Schisandra chinensis* fruit (p.o.)	*S. chinensis* fruit extract increased AUC and Cmax of orally administered midazolam, but there was no little change in the PK properties of intravenously administered midazolam in rats	Due to intensive inhibitory effect on CYP3A in intestine, not in liver	[64]
**Dose**	*Andrographis paniculata* extract (p.o.)	Low dose of Andrographis Herba extract increases theophylline elimination, whereas high-dose of *A. paniculata* extract decreases theophylline elimination	Due to induction of CYP1A2 by low-dose treatment of *A. paniculata* extract, but inhibition of CYP1A2 by its high-dose treatment	[72]
	*Tinospora cordifolia* aqueous-alcoholic extract (p.o.)	High-dose of *T. cordifolia* aqueous-alcoholic extract, not low-dose, reduced the clearance and increased bioavailability of glibenclamide, respectively, in rats	Due to the inhibition of CYP2C9, 2D6 and 3A4 in liver by high-dose of *T. cordifolia* aqua-alcoholic extract, not low-dose	[73]
**Treatment period**	*S. chinensis* fruit extract (p.o.)	Long-term treatment of *S. chinensis* fruit extract reduced AUC and Cmax of orally administered midazolam, but the AUC and Cmax of orally administered midazolam were increased after single treatment of Schisandrae Chinensis Fructus extract	Due to stronger induction of CYP3A in liver and intestine than inhibition of CYP3A in long-term treatment; Due to stronger inhibition of CYP3A in intestine, not in liver after single treatment	[72]
	*G. biloba* leaf extract (p.o.)	Single treatment of *G. biloba* leaf extract increased the intravenously administered diltiazem concentrations in plasma, but long-term treatment of ginkgo biloba leaf extract reduced the intravenously administered diltiazem concentrations in plasma	Due to inhibition of CYP3A in liver in singe treatment of *G. biloba* leaf extract; due to induction of CYP3A in liver after long-term treatment	[68,76]
	*Lonicera japonica* extract (p.o.)	28-day treatment of *L. japonica* extract increased metformin concentration in liver along with the enhancement of glucose tolerance activity of metformin, but single and 7-day treatment of *Lonicera japonica* extract did not alter metformin concentration in plasma and liver as well as glucose tolerance activity.	Due to reduction in mate1-mediated biliary excretion of metformin by 28-day treatment of Lonicera japonica extract	[29]
	*Houttuynia cordata* extract (p.o.)	28-day treatment of *H. cordata* extract increased metformin concentration in plasma, liver and kidneys along with the enhancement of glucose-lowering effect in rats, but there no change of PK and PD of metformin after single and 7-day treatment of *Houttuynia cordata* extract	Increase in metformin plasma concentrations due to the decrease in renal oct2-mediated renal excretion of metformin and metformin concentration in kidneys; enhancement of glucose tolerance activity due to the increase in oct1-mediated renal uptake of metformin	[30]

## Data Availability

Data for this review is publicly available through previously published manuscripts as referenced.
