# Erratum: Choi, Y.H., et al. Multifaceted Factors Causing Conflicting Outcomes in Herb–Drug Interactions. *Pharmaceutics* 2021, *13*, 43

**DOI:** 10.3390/pharmaceutics13030337

**Published:** 2021-03-05

**Authors:** Young Hee Choi, Young-Won Chin

**Affiliations:** 1 College of Pharmacy and Integrated Research Institute for Drug Development, Dongguk University_Seoul, 32 Dongguk-lo, Ilsandong-gu, Goyang-si, Gyeonggi-do 10326, Korea; 2 College of Pharmacy and Research Institute of Pharmaceutical Sciences, Seoul National University, Seoul 08826, Korea; ywchin@snu.ac.kr

The authors wish to make the following correction to the funding information [1]: “NRF-2018R15A2023217 (Y.H.C.)” should be “NRF-2018R1A5A2023127 (Y.H.C.)”. The updated “Funding” section is: This research was funded by the National Research Foundation of Korea (NRF), grants were funded by the Korean Government (MSIT) (NRF-2016R1C1B2010849 and NRF-2018R1A5A2023127 (Y.H.C.) and NRF-2019R1A2C2009053 (Y.-W.C.)).

The authors would like to apologize for any inconvenience caused to the readers by these changes.
